# Application of Ultrasonic Guided Waves for Inspection of High Density Polyethylene Pipe Systems

**DOI:** 10.3390/s20113184

**Published:** 2020-06-03

**Authors:** Premesh Shehan Lowe, Habiba Lais, Veena Paruchuri, Tat-Hean Gan

**Affiliations:** 1 Nuclear AMRC, University of Sheffield, Brunel Way, Catcliffe, Rotherham S60 5WG, UK; shehan.lowe@namrc.co.uk; 2 Brunel Innovation Centre, Granta Park, Great Abington, Cambridgeshire CB21 6AL, UK; habiba.lais@brunel.ac.uk; 3 University of Surrey, Stag Hill, Guildford GU2 7XH, UK; paruchuriveena@hotmail.com

**Keywords:** thermoplastic pipe, ultrasonic guided waves, inspection, array design, non-destructive testing, finite element analysis, industrialization, flexible macro-fiber composite transducer

## Abstract

The structural integrity assessment of thermoplastic pipes has become an interesting area of research due to its elevated usage in the liquid/gas transportation industry. Ultrasonic guided wave testing has gained higher attention from industry for the inspection of elongated structures due to the reduced inspection time and cost associated with conventional non-destructive testing techniques, e.g., ultrasonic testing, radiography, and visual inspection. Current research addresses the inspection of thermoplastic pipes using ultrasonic guided waves as a low cost and permanently installed structural health-monitoring tool. Laboratory and numerical investigations were conducted to study the potential of using ultrasonic guided waves to assess the structural health of thermoplastic pipe structures in order to define optimum frequency range for inspection, array design, and length of inspection. In order to achieve a better surface contact, flexible Macro-Fiber Composite transducers were used in this investigation, and the Teletest^®^ Focus+ system was used as the pulser/receiver. Optimum frequency range of inspection was at 15−25 kHz due to the level of attenuation at higher frequencies and the larger dead zone at lower frequencies due to the pulse length. A minimum of 14 transducers around the circumference of a 3 inch pipe were required to suppress higher order flexural modes at 16 kHz. According to the studied condition, 1.84 m of inspection coverage could be achieved at a single direction for pulse-echo, which could be improved by using a higher number of transducers for excitation and using pitch-catch configuration.

## 1. Introduction

The use of thermoplastic pipe systems, i.e., high density polyethylene (HDPE), are becoming increasingly popular for many applications, such as transporting gas, water, and chemicals. The corrosion and high fouling resistance as well as the lightweight properties of thermoplastic pipes, makes it more attractive for the industry to replace the usage of steel pipes in some applications with safety-critical infrastructures, for example, in nuclear power stations [1,2]. 

The material degradation over time is a common problem in pipe networks, as it causes loss of structural integrity, which can lead to severe catastrophic failures. For example, the two largest water utilities in the world, Veolia environment and Suez environment, experienced severe HDPE pipe system failures due to degradation that caused a premature failure by chemically induced embrittlement [3]. Veolia and Suez also examined samples of HDPE pipes in service around the world under various pressures, temperatures, and disinfectant regimes, and findings revealed that the pipes are subject to degradation and pose a risk of premature failure, particularly in high temperature environments. The degradation can occur due to adverse environmental effects, mechanical damage, or manufacturing defects [4,5]. Early detection of such degradation is important to mitigate catastrophic failures. Furthermore, due to the complexity of pipeline infrastructures, particular regions in the pipe are inaccessible, and when in operation, it is almost impractical to inspect manually. Therefore, it is important to develop an inspection technique for thermoplastic pipe systems to assess the structural health of in-situ conditions [1,2].

The current state-of-the-art inspection techniques for the inspection of HDPE pipes include localized non-destructive testing (NDT) techniques such as radiography, conventional ultrasonic testing, acoustic emission, visual inspection, thermal imaging, laser scanning, and ground penetrant testing [6,7]. The current paper studies the potential use of ultrasonic guided waves (UGWs) as a low cost and permanently installed monitoring tool for the inspection of HDPE pipe systems.

A great deal of research was conducted on the application of UGWs to inspect metallic structures, and it has gained a high level of attention from industries due to their long-range inspection capabilities from a single test location (~100 m of pipelines from single test location) [8]. However, the application of UGW to inspect non-metallic structures, i.e., HDPE pipes, is still in its infancy. UGW based techniques allow one to inspect elongated structures such as pipelines from a single test location [9]. Different transduction configurations can be used to excite UGW on structures; commonly used configurations are piezoelectricity and magnetostriction. There is a large interest in piezoelectric transducers due to their low cost and wide availability [10].

Flexible macro-fiber composite (MFC) transducers were used to design the transducer array to achieve a better contact with the structure of interest compared to the brittle piezoelectric transducers used in previous studies [11]. The present authors studied the benefit of the use of flexible transduction to achieve a rigid contact for UGW application with a view to improve the signal-to-noise ratio [12]. Key parameters studied in this investigation were optimum number of transducers around the pipe circumference, optimum operating frequency range, and length of propagation. 

The paper is organized as follows. Section 2 reviews the state-of-the-art inspection of thermoplastic pipes and presents the theoretical background. The laboratory experiment conducted in this investigation is reported in Section 3. Numerical analysis of the UGW propagation in thermoplastic pipes is documented in Section 4. Results and discussions can be found in Section 5, followed by conclusions and further work in Section 6.

## 2. Theoretical Background

### 2.1. Elastic Waves in Solid Media

An understanding of elastic wave propagation within solid media is important for the development of inspection procedures. The wave propagation in elastic infinite media is presented in literature and is briefly outlined [13]. Navier’s equation of motion for an isotropic elastic unbounded media is shown in Equation (1):

(1) $$\left(\lambda +\mu \right)\nabla \left(\nabla \cdot u^{\prime }\right)+\mu \nabla^{2}u^{\prime }=\rho \frac{\partial^{2}u^{\prime }}{\partial t^{2}}$$

where λ and μ are Lamé constants, $u^{\prime }$ is the displacement vector, $\nabla^{2}$ is the Laplace operator, and ρ is the material density. Helmholtz decomposition can be used to write $u^{\prime }$ as a sum of the compressional scalar potential, Ø, and an equivoluminal vector potential, Φ,

(2) $$u^{\prime }=\nabla Ø+\nabla x\Phi$$

with,

(3) ∇×Φ=0

By substituting the potentials of Helmholtz decomposition (Equations (2) and (3)) into Navier’s equation (Equation (1)) of motion, one receives two separate formulae for the unknown potentials that govern longitudinal waves (Equation (4)) and shear waves (Equation (5)).

(4) $$\left(\frac{\partial^{2}Ø}{\partial t^{2}}\right)=c_{l}^{2}\nabla^{2}Ø$$

(5) $$\left(\frac{\partial^{2}\Phi }{\partial t^{2}}\right)=c_{s}^{2}\nabla^{2}\Phi$$

where $c_{l\;}$ and $c_{s}$ are the velocities of longitudinal and shear waves, which can be written as,

(6) $$c_{l\;}=\sqrt{\frac{\lambda +2µ}{\rho }}$$

(7) $$c_{s\;}=\sqrt{\frac{µ}{\rho }}$$

Based on this, there are two types of elastic waves that can propagate within solids, known as longitudinal waves (Equation (6)) and shear waves (Equation (7)). These two modes can travel in any direction in an infinite solid media. However, UGWs are constrained to propagate along the axis of the structure and therefore result in many possible modes of vibration. 

Depending on the waveguide geometry, the material properties, and the excitation frequency of the discrete pulse, the potential number of wave modes varies [8]. Due to this complexity, a nomenclature was defined for identification of UGW modes in cylindrical structures. The nomenclature used throughout this study was proposed by Meitzler [14] and popularized by Silk and Bainton [15]. Conferring to this nomenclature, the vibrational modes in cylindrical structures are based on the following format, X(n,m), where X represents the character to denote whether the vibration modes are longitudinal and axisymmetric (L), torsional and axisymmetric (T), or non-axisymmetric (also shown as flexural) (F). The n is a positive integer giving the identification of harmonic variations of displacement around the circumference, and m is a positive integer to indicate the incremental order of the modes of vibration within the wall. For a given geometry, the number of UGW modes that can be excited at a given frequency can be calculated using dispersion curves. 

**Figure 1:**
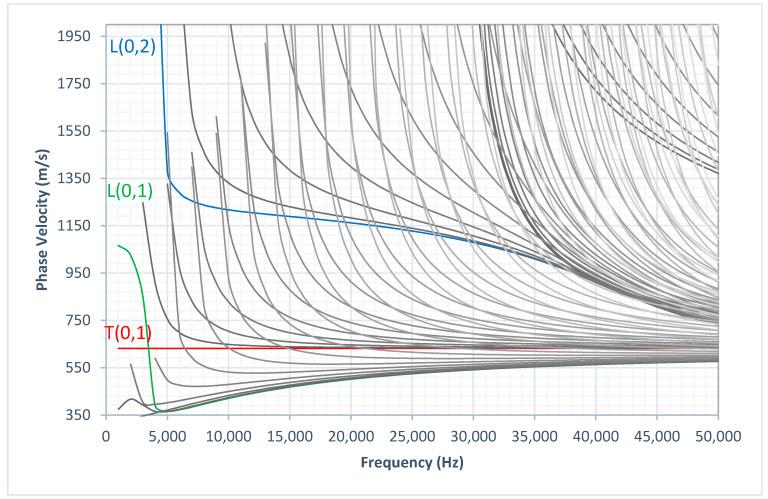
Phase velocity dispersion curves of a 3 inch diameter, 10 mm thick HDPE pipe.

**Figure 2:**
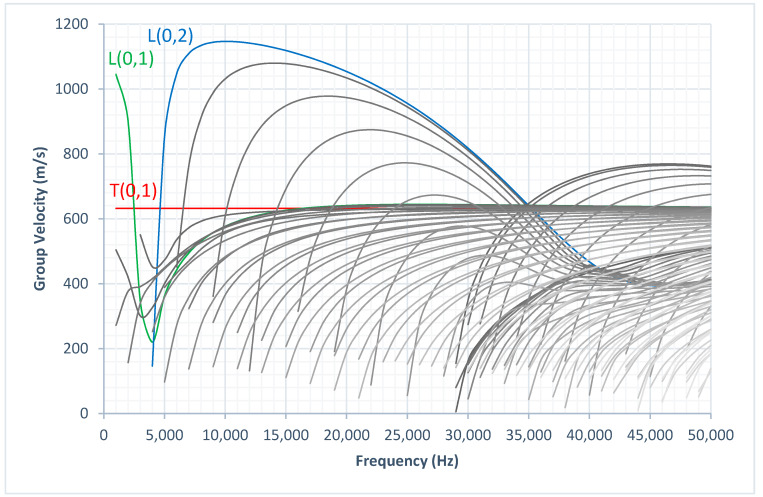
Group velocity dispersion curves of a 3 inch diameter, 10 mm thick HDPE pipe.

**Figure 3:**
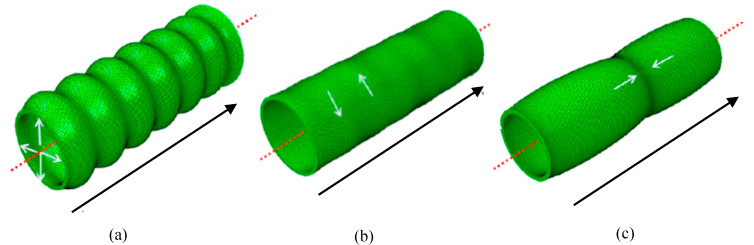
Displacement patterns of the axisymmetric modes (**a**) L(0,1) (**b**) T(0,1), and (**c**) L(0,2). The black arrow represents the direction of propagation, and the red dashed line represents the central axis.

Figure 1 illustrates the phase velocity dispersion curves and Figure 2 illustrates the group velocity dispersion curves calculated using graphical user interface for guided ultrasonic waves (GUIGUW) for a 3 inch diameter, 10 mm wall thickness HDPE pipe [16] based on the material properties listed in Table 1. There were three axisymmetric modes that could be excited at the UGW operating frequency range noted as: L(0,1), T(0,1), and L(0,2). The displacement pattern of these three modes are illustrated in Figure 3. As illustrated in Figure 3, the T(0,1) wave mode has a circumferential displacement, the L(0,2) wave mode has a mainly axial displacement, and the L(0,1) wave mode has both radial and axial displacement. 

### 2.2. Thermoplastic Pipe Inspection

Thermoplastic pipe inspection using UGW is an arising area of interest. Ghazali et al. used a piezoelectric stack actuator for excitation of UGW to detect cracks in plastic pipes [1]. Different pipe specimens such as un-cracked pipes and pipes with different number of cracks were investigated. Each pipe specimen was tested with different distances between the transmitter and receiver and at different excitation frequencies; 0.5 kHz, 3 kHz, 6 kHz, and 9 kHz. Results showed differences in frequency response between un-cracked and cracked pipes and also between the numbers of cracks, proving that UGW can be used to detect cracks in plastic pipes. However, the monitored amplitude over the frequency range decreased in all samples due to the attenuation in the medium as the distance from the source increased and had a higher dead-zone. 

Zhu et al. showed how a water-coupled focused conventional ultrasonic transducer can be used to study the effect of ground conditions surrounding the pipe. This study contains the interaction with pipe material, soil, fluids, and formation of voids in the soil bedding. Two common plastic pipe materials; polyvinyl chloride (PVC) and HDPE, were tested [17]. Detection of crack, material loss in HDPE plate, and location of voids external to pipe wall were investigated. Ultrasound scanning showed that the method was effective in detecting and locating various sized grooves, slots, cracks, and voids in various soil conditions.

Recent developments on nonlinear ultrasonic behavior such as harmonic generation, nonlinear resonance, and mixed frequency response have shown potential in detecting a crack at its preliminary stage. The nonlinear ultrasonic technique can detect a crack by the harmonics and the modulations created by a nonlinear source i.e., the defect. When an ultrasonic wave at a single input frequency is propagated through the nonlinear source, additional harmonics of the input frequency are generated. Also, when two ultrasonic waves at two distinct frequencies are propagated, the interaction caused between them produces a nonlinear response. An amplitude modulation signal can be produced between two signals by addition of the amplitude of each signal. Hong et al. used piezoelectric transducers for UGW excitation based on nonlinear amplitude modulation to study crack detection in a PVC pipe [18]. Eleven different damaged states with different crack lengths (0–35 mm) and depths (0–2.5 mm) were studied. A decrease in energy of the response signal was found as the crack grew and the difference was more discernible with a high frequency ultrasonic signal of 165 kHz than the low frequency ultrasonic signals in the range of 6–16 kHz. However, the preliminary states of damage were hard to distinguish at all frequencies of ultrasound. It requires data processing to amplify the signal in order to see the difference between the states.

Moreover, research on damage detection in plastic structures has been conducted using various methods. For instance, Cheraghi et al. investigated the detection of damage in adhesively bonded joints in plastic pipes and compared the effectiveness of different methods based on Fourier transform, continuous wavelet transform, and Hilbert Huang transform [19]. Nevertheless, the detection methods in plastic pipes mentioned are mainly aimed at detecting gross cracks or leakage, which may already cause losses before being detected. In some critical plastic pipe applications, such as caustic substance transport pipelines, these methods are obviously not sufficient, since in these cases, once leakage happens, it often leads to factory shutdown or even serious personal injury. Furthermore, a crack growing to a critical point at an alarming rate can lead to catastrophic consequences without any sufficient warning [20]. Thus, preliminary crack detection on plastic pipelines is demanded in these cases to avoid human injury and asset loss. This concludes the need for a robust and cost effective tool to monitor the structural health of thermoplastic pipes such as HDPE pipes used in power industry for gas/liquid transportation. 

## 3. Laboratory Experiments

### 3.1. Experimental Set-Up

**Figure 4:**
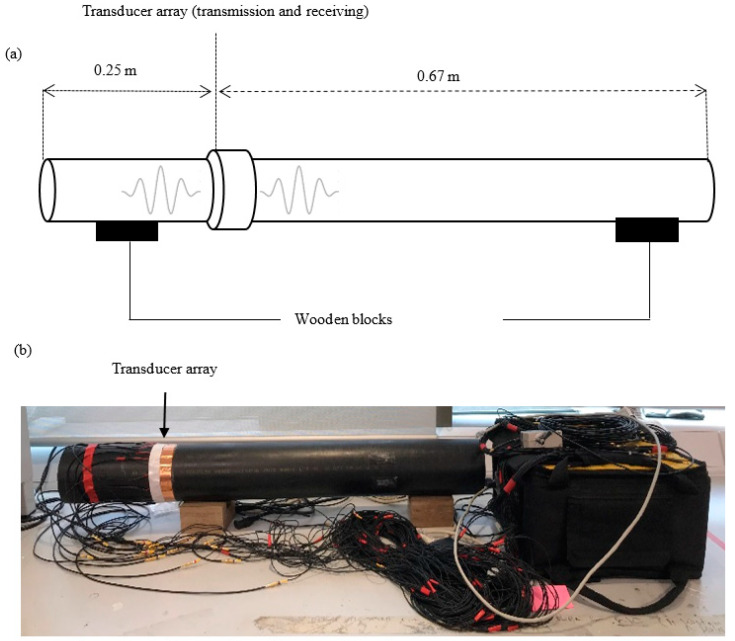
Laboratory experimental setup: (**a**) schematics; (**b**) setup.

Prior to designing the optimum transducer array for the UGW inspection of HDPE pipes, it is important to study the UGW propagation; therefore, laboratory experiments were conducted to study the UGW propagation on a 0.92 m long, 3 inch diameter, 10 mm thick HDPE pipe section. The test sample rested on two wooden blocks to minimise UGW interaction with its surroundings. The experimental set-up is illustrated in Figure 4. To determine an approximation of the minimum number of transducers required around the circumference of the pipe specimen, Equation (8) calculates this approximation based on the highest order of flexural mode and dependent on the desired operating frequency. Based on the group velocity dispersion curves presented in Figure 2 and as suggested by Rose in 2014 [13], Equation (8) is formulated to calculate the minimum number of transducers (16 in this study) needed to excite axisymmetric longitudinal modes, L(0,1), L(0,2), and suppress excitation of flexural modes and reduce signal noise.

(8) $$N_{Tx}=Max_{fm}+1$$

where $N_{Tx}$ is the number of transducers and $Max_{fm}$ is the highest order of flexural mode in the desired operating frequency. 

Flexible MFC2814 macro fiber composite transducers were used in the present study to obtain a higher surface contact between the transducer and the pipe surface [21,22]. Due to the dimensions of transducers, up to 14 transducers were bonded around the circumference on the test sample, which can be improved by transducer miniaturization in future studies. 

The state-of-the-art commercially available Teletest^®^ Focus+ system was used to excite the transducers. The data acquisition was conducted in the pulse-echo configuration due to the length limitation of the pipe sample. A frequency sweep was conducted from 10 to 30 kHz in 1 kHz increments. A 5-cycle Hann-windowed excitation pulse was applied for a discrete input excitation signal. Using the velocities of the two longitudinal modes, L(0,1) and L(0,2), obtained from the group velocity dispersion curves, the expected time-of-arrival (ToA) at possible reflections are tabulated in Table 2 for a 16 kHz operating frequency.

### 3.2. Experimental Results

**Figure 5:**
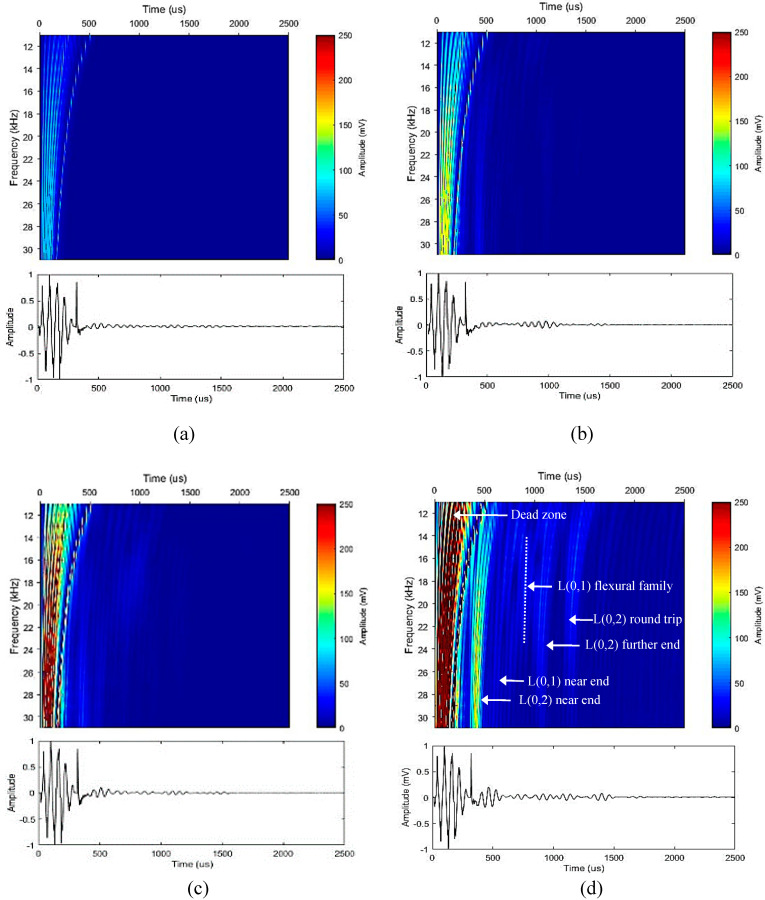
Experimental results over a range of frequencies for different transducer configurations and its corresponding time-domain results at 16 kHz: (**a**) one transducer; (**b**) two evenly spaced transducers; (**c**) four evenly spaced transducers; (**d**) eight evenly spaced transducers.

**Figure 6:**
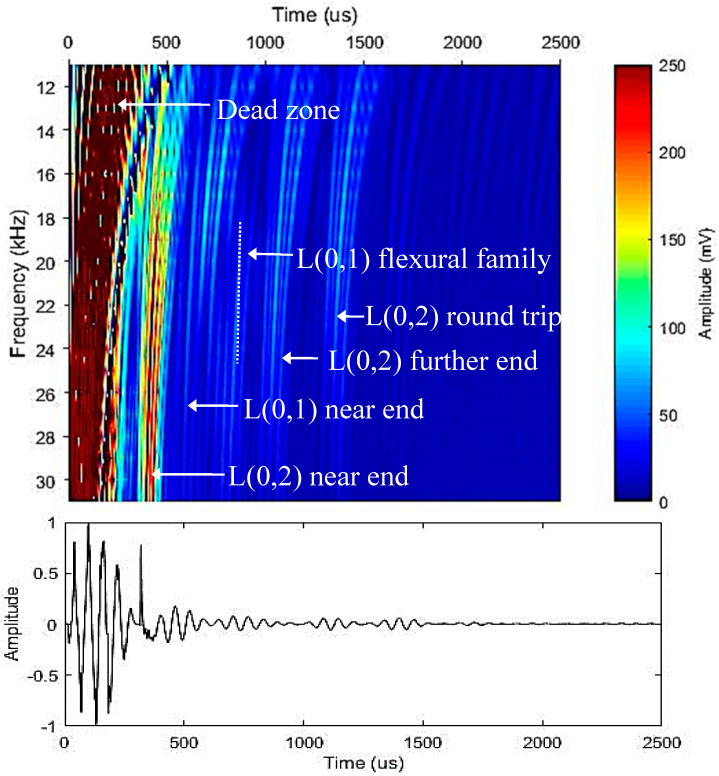
Experimental results over a range of frequency for excitation using 14 transducers and its corresponding time-domain results at 16 kHz.

Figure 5 and Figure 6 illustrates the surface plot for the acquired data over a frequency range for different transducer configurations. It is evident that having a low number of transducers is not sufficient due to the excitation of higher order modes causing noise within the signal. A higher number of transducers leads to obtaining a higher inspection coverage by suppressing the transmission of higher order modes. ToA results correspond to each reflection tabulated in Table 2. As expected, the first mode to arrive was the near end reflection of L(0,2) followed by the near end reflection of L(0,1). After that, the L(0,1) mode dissipated over time due to the higher level of attenuation [23]. Then came the further end reflection of L(0,2) followed by its round trip covering 1.84 m of propagation. All the reflections were identified accordingly and labeled to ease the data interpretation. 

**Figure 7:**
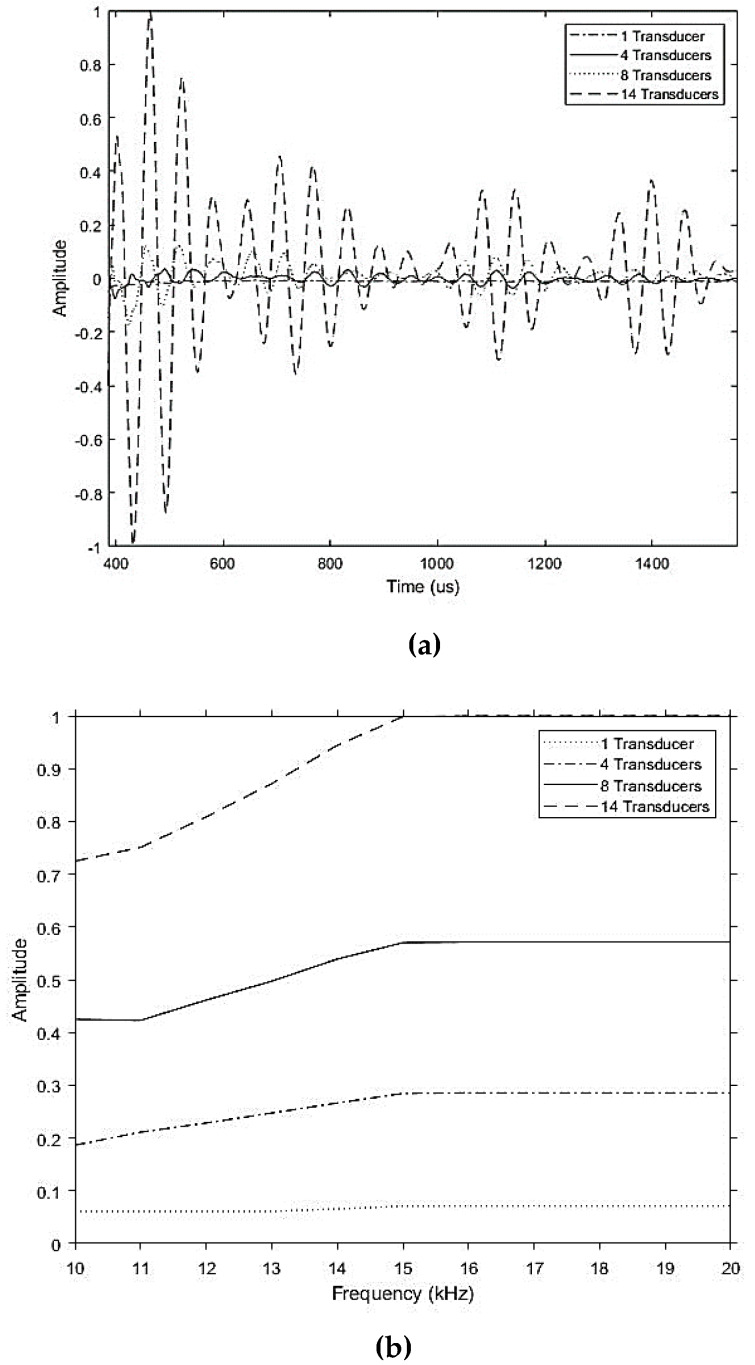
Experimental results over (**a**) time and (**b**) range of frequency for excitation at a different number of transducers, illustrating the incremental amplitude increase when the number of transducers increased.

Figure 7 shows that th increase in number of transducers excited in the longitudinal wave mode; increases the amplitude across the operating frequencies, as well as improvements in the signal visibility from the noise. The number of higher order modes related to each family of fundamental modes for a range of frequencies is tabulated in Table 3. A minimum of 16 transducers was required in order to suppress the excitation of higher order modes below 20 kHz for the inspection of HDPE pipes. There were higher order flexural modes excited over 14 kHz for the eight transducer configuration and over 18 kHz for the 14 transducer configuration. This corresponded to the flexural family of L(0,1) mode after F(12,1).

## 4. Numerical Analysis

### 4.1. Finite Element Model

To assist in understanding the wave propagation over the HDPE pipe sample, a finite element analysis (FEA) was conducted in COMSOL Multiphysics 5.3. The model consisted of a 0.92 m length HDPE pipe with a 110 mm outer wall diameter and an 88 mm inner wall diameter. The material properties can be found in Table 1, and the layout of the FEA is illustrated in Figure 4. 

The transmission/receiving location was placed 0.25 m from the pipe end to replicate the configuration in the laboratory trials (refer to Figure 4). 

The transducer configuration was represented as point loads in the FEA model. Symmetry was used within the model to reduce computational time by exciting one-point load. The point load was applied parallel to the length of the pipe to generate the longitudinal wave mode. The pressure point was a sine wave modulated using Hann window function as shown below:

(9) $$U\left(t\right)=\frac{1}{2}\sin\left(2\pi ft\right)\left[1-\left(\frac{2\pi ft}{n}\right)\right]$$

For the dynamic transient simulation to map out the wave propagation throughout the structure, the mesh was required to be calculated to an optimal mesh element length. The wave equation required the meshing to compliment the time stepping within the solver to produce an accurate solution. The mesh sizing required a minimum of eight second order mesh elements per wavelength. To calculate the maximum allowed element size (*h_o_*), the following equation was used:

(10) $$h_{o}=\frac{c}{Nf_{o}}\;\;\;\;\;$$

where *c* is the velocity, *N* is the number of elements per wavelength, and *f_0_* is the center frequency.

Using the material properties found in Table 1, the velocity was calculated to be 1076 m/s and was used to calculate the element size (Equation (10)) based on 15 elements per wavelength at the selected operating center frequency of 16 kHz, found from the experimental investigation. 

The COMSOL model investigates the longitudinal wave mode at different numbers of transducers and at a different number of cycles. The operating frequency was kept constant at 16 kHz, as selected from the laboratory trials. To compare the receiving signal, the ToA was calculated using the group velocity of the longitudinal wave mode found in Figure 2. The equation to calculate ToA is as follows:

(11) $$Time\;of\;Arrival=\frac{x}{c_{l}}$$

where *x* is the distance from transmitter to receiver, and *c_l_* is the group velocity of the longitudinal wave mode at the operating frequency.

### 4.2. Parametric Study

The FEA model was used to carry out a parametric study to investigate the number of evenly spaced transducers excited within a configuration (1, 4, 8, 14, and 20) by utilizing the selected optimal frequency found in the laboratory investigation. The numerical model was used to optimize the number of transducers within the array to cancel any excitation due to higher order modes (axisymmetric wave modes). The selected optimal transducer array was then used to investigate the number of cycles of the discrete pulse (3, 5, and 10 cycles) to select the optimal cycle input for inspection to allow separation of the receiving pulse. The separation of the signal is most important at joints and bends, as this needs to be identifiable as a feature during inspection. 

## 5. Results and Discussion

**Figure 8:**
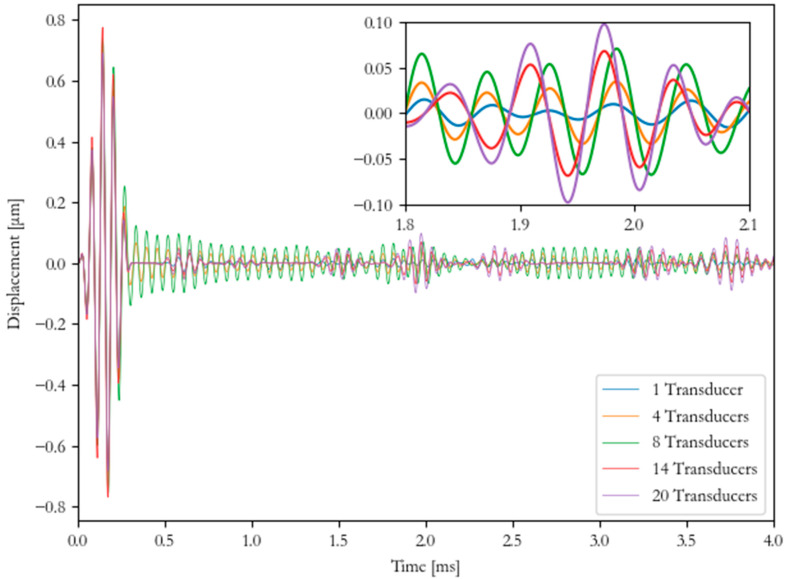
Numerical results of different numbers of transducers excited at 16 kHz in time-domain.

A point load was used to excite the discrete pulse, and a monitoring point at the excitation location was used to collect the pulse-echo signal for each FEA computation, as shown in Figure 8. When comparing the signal, the 1 and the 4 transducer configurations had a low amplitude, as the configuration was not producing enough energy to propagate. The 8 transducer configuration produced a lot of noise in the signal (approximately ±0.05 µm) due to producing multi-modal responses, as the configuration was not optimized to generate pure longitudinal mode. The 14 and the 20 transducers produced a high amplitude signal that had very little noise (approximately ±0.002 µm). The dispersion curve shows that approximately 14 transducers were required on the circumference to generate pure longitudinal modes. 

The frequency range of the pulse is called the bandwidth, and the following equation is used to define the frequency range of the bandwidth:

(12) $$f_{bw}=f\pm \frac{\left(k_{i}+2\right)f}{n}$$

where *f_bw_* is the frequency bandwidth, and *k_i_* is the bandwidth of the desired lobe, where *k_i_* = 0 is for the main lobe and *k_i_* = 1 is the side lobe.

The frequency range for each investigated cycle input has been calculated and tabulated in Table 4. The selection of the most suitable number of cycles within the discrete pulse for UGW inspection was critical. This was due to a higher number of cycles within the pulse resulting in a smaller frequency bandwidth and longer pulse length, whereas fewer cycles resulted in a wider frequency bandwidth and shorter pulse length. A wider frequency bandwidth can be problematic by causing dispersion, especially if the velocities of the chosen mode change significantly over the frequency.

When comparing each transducer configuration at the received signal, there was a phase shift in the signals due to the amplitude and the multi-mode response. 

**Figure 9:**
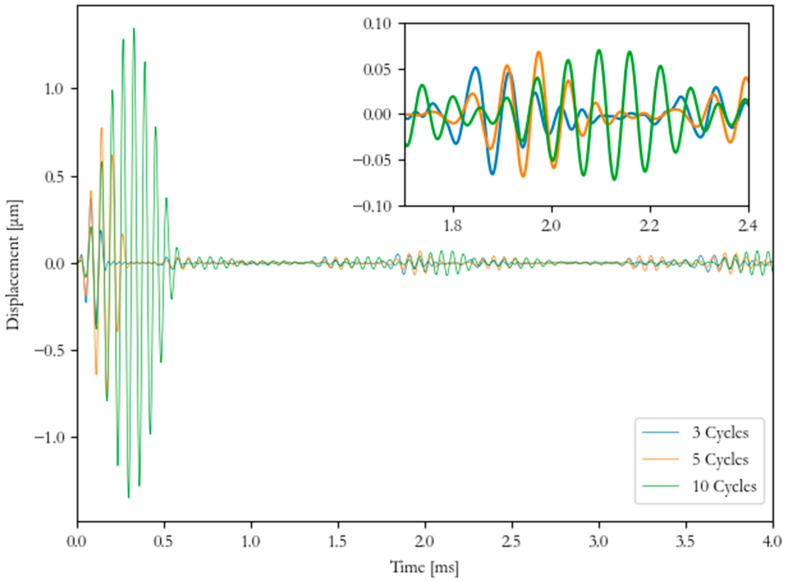
Numerical results of 14 transducer case excited at 16 kHz at a different number of cycles in time-domain.

The 14 transducer case was selected to investigate the number of cycles. Figure 9 shows that the 3 cycle case did not generate a clear signal when receiving the pulse. The 10 cycle case generated a signal that could be detected; however, due to the length of the pulse, this spread over time, which could be difficult for data interpretation. For the studied case, 5 cycles allowed the signal to be detected and interpreted. 

## 6. Conclusions and Further Work

In the present study, authors conducted a series of experimental cases and numerical validations to understand the potential of using UGWs for the inspection of HDPE pipes, which are mainly used in the energy sector for gas transportation. The mode of interest in this case was the second order longitudinal UGW mode. An array of 14 transducers was used in this study, which was governed by the size of the transducers and, according to the dispersion curves calculated, it required at least 14 transducers to suppress flexural modes. At the conditions studied, 1.84 m inspection coverage was achieved at pulse configuration. This could be improved by increasing the number of transducers around the circumference and using pitch-catch configuration for data acquisition. A numerical model was built using COMSOL Multiphysics based on the experimental trials, which was then used to conduct a parametric study to optimize the operational parameters, i.e., number of transducers and number of cycles. Such a model helps the current authors and other researchers interested in this topic to conduct their investigations without carrying out time and cost heavy experiments. The next stage for this research is to apply the validated numerical modeling for investigation of defects within the pipeline and its joints, such as delamination and dis-bond, allowing further analysis of signal responses by the presence of a defect. Such a study will provide confidence of defect characterization and sizing for industrial adaptation. Further research will also be conducted on the miniaturization of the UGW flexible transducers to accommodate a higher number of transducers around the circumference, which will increase the inspection resolution. 

## Figures and Tables

**Table 1:** Assumed material properties in the numerical simulation. HDPE: high density polyethylene.

Material Property	HDPE
Young’s Modulus	1.1 GPa
Poisson’s Ratio	0.45
Density	950 kg/m^3^

**Table 2:** Calculated time-of-arrival (ToA) for the expected reflections from pipe ends at 16 kHz operating frequency.

Reflection (m)	Expected ToA (µs)	
	L(0,1)	L(0,2)
0.5	803.57	444.05
1.34	2150.00	1190.05
1.84	2953.50	1634.10

**Table 3:** Number of flexural modes corresponded to each family of fundamental modes over a range of frequencies.

Frequency (kHz)	Number of Higher Order Modes		
	T(0,1)	L(0,1)	L(0,2)
10	4	7	2
12	5	8	2
14	6	9	3
16	7	10	3
18	8	11	4
20	9	12	5

**Table 4:** Number of cycles studied and its representative frequency bandwidth.

Number of Cycles	Frequency Bandwidth [kHz]	
	Main Lobe	Side Lobe
3	5.33–26.67	0–32
5	9.6–22.4	6.4–25.6
10	12.8–19.2	11.2–20.8
